# An electrophysiologist’s guide to dorsal horn excitability and pain

**DOI:** 10.3389/fncel.2025.1548252

**Published:** 2025-04-02

**Authors:** Iván Rivera-Arconada, Mark L. Baccei, José A. López-García, Rita Bardoni

**Affiliations:** ^1^Department of Systems Biology, University of Alcala, Madrid, Spain; ^2^Department of Anesthesiology, Pain Research Center, University of Cincinnati, Cincinnati, OH, United States; ^3^Department of Biomedical, Metabolic and Neural Sciences, University of Modena and Reggio Emilia, Modena, Italy

**Keywords:** spinal cord, nociception, electrophysiological techniques, ionic channels, action potentials, dorsal horn neurons, firing pattern

## Abstract

The dorsal horn of the spinal cord represents the first site in the central nervous system (CNS) where nociceptive signals are integrated. As a result, there has been a rapid growth in the number of studies investigating the ionic mechanisms regulating the excitability of dorsal horn neurons under normal and pathological conditions. We believe that it is time to look back and to critically examine what picture emerges from this wealth of studies. What are the actual types of neurons described in the literature based on electrophysiological criteria? Are these electrophysiologically-defined subpopulations strongly linked to specific morphological, functional, or molecular traits? Are these electrophysiological properties stable, or can they change during development or in response to peripheral injury? Here we provide an in-depth overview of both early and recent publications that explore the factors influencing dorsal horn neuronal excitability (including intrinsic membrane properties and synaptic transmission), how these factors vary across distinct subtypes of dorsal horn neurons, and how such factors are altered by peripheral nerve or tissue damage. The meta-research presented below leads to the conclusion that the dorsal horn is comprised of highly heterogeneous subpopulations in which the observed electrophysiological properties of a given neuron often fail to easily predict other properties such as biochemical phenotype or morphology. This highlights the need for future studies which can more fully interrogate the properties of dorsal horn neurons in a multi-modal manner.

## 1 Introduction

**FIGURE 1:**
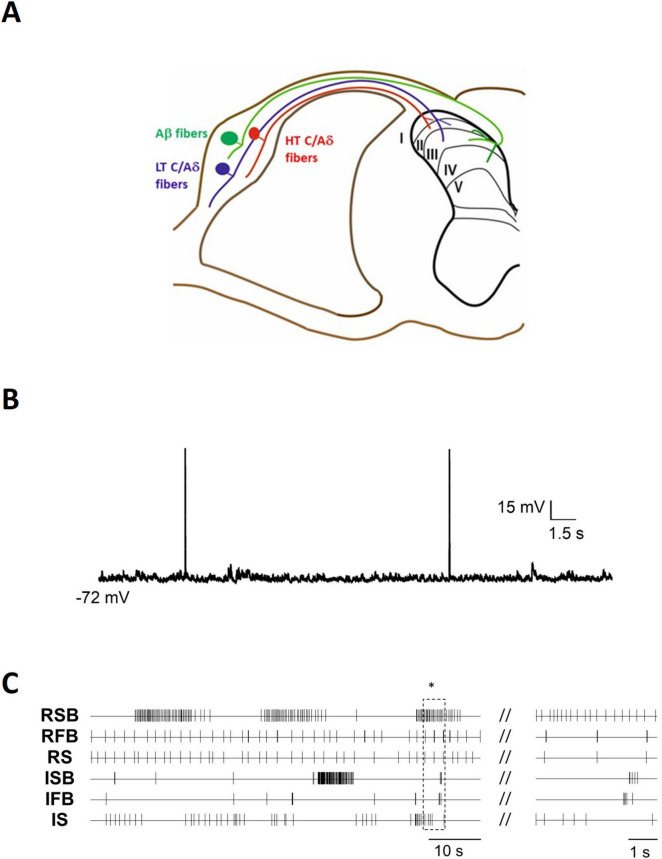
Spontaneous activity exhibited by dorsal horn neurons. **(A)** Schematic representation of the spinal cord dorsal horn and the primary afferent projections to the different laminae. **(B)** Representative example of irregular spontaneous firing recorded in an adult mouse lamina I spinoparabrachial (projection) neuron in an *ex vivo* spinal cord preparation. **(C)** Extracellular recordings were obtained in an *ex vivo* slice from a neonatal mouse using a multielectrode array situated in the superficial laminae of the cord. Action potentials of each individual neuron are represented as vertical lines and include representative examples of 6 neurons with different patterns recorded simultaneously. The right panel shows the area indicated by the asterisk in an expanded time base. Firing patterns include both regular and irregular patterns, with spikes in isolation as well as grouped in burst (RSB, regular slow burst; RFB, regular fast burst; RS, regular simple; ISB, irregular slow bursts; IFB, irregular fast burst; IS, irregular simple firing).

The spinal cord dorsal horn (DH) serves as the initial integration site where somatosensory input is processed by the central nervous system. It is organized into distinct laminae, each associated with specific types of sensory information. Primary afferent fibers (PAFs) that transmit pain, touch, itch, and proprioceptive inputs are categorized into different types (Aα, Aβ, Aδ, and C), each conveying distinct sensory modalities (Figure 1A). In particular, myelinated Aβ and low threshold (LT) Aδ fibers, which mediate innocuous mechanical sensitivity, primarily project to lamina II inner, III, and IV. In contrast, high threshold (HT) myelinated Aδ and unmyelinated C fibers, which mediate pain, thermal sensations, and itch, mainly terminate in lamina I and II (for review see Todd, 2010; Merighi, 2018). Low-threshold mechanoreceptive fibers also include a specific group of C fibers that project to lamina II. These fibers transmit pleasant touch and modulate unpleasant mechanical pain sensations (Liu et al., 2007; Seal et al., 2009). Within DH laminae, PAFs synapse onto both excitatory and inhibitory local interneurons as well as projection neurons, that extend their axons to supraspinal centers. In response to sensory stimulation, DH neurons may generate action potentials (APs) with varying firing patterns. In addition to stimulus-evoked activity, some DH neurons exhibit spontaneous firing. DH neuron excitability is shaped not only by sensory input but also by intrinsic electrical properties.

In this review, we provide an overview of early and recent data related to the generation and modulation of DH neuronal activity. In particular, we will review the mechanisms underlying spontaneous and evoked AP firing in DH neurons. Additionally, we will explore the intrinsic membrane properties of specific DH neuron populations and discuss how chronic pain can lead to modifications in neuronal excitability within spinal nociceptive circuits. While dorsal horn neurons can be classified via a multitude of other properties such as their transcriptional profile and functional role in somatosensation, these valid and informative classification approaches nonetheless fall outside the scope of the present review. Instead, we selectively focus on the electrophysiological signatures of dorsal horn neurons, based on the belief that further improving our understanding of the mechanisms regulating DH neuron excitability is essential for comprehending how DH networks operate under normal and pathological conditions.

## 2 Overview of electrophysiological and complementary methods to study dorsal horn neurons

Several different technical approaches have been used to gather information on the electrical behavior of spinal cord neurons and their sensory coding capabilities. These include a number of different animal preparations, recording techniques and stimulation protocols.

*In vivo preparations* of cats and rats coupled to extracellular single electrode techniques were commonly used in the past century and produced very useful data regarding how neurons respond to natural stimulation. Single neurons were recorded, one at a time with glass micropipettes or metallic electrodes, and their receptive fields explored. These experiments served to define the main functional types of DH neurons and to explore their general response properties thoroughly. Other *in vivo* preparations included different methods of spinalization to enable studies on descending modulation and in some exceptional cases, recordings were obtained from awake animals to check the validity of results obtained with anesthetized animals (Herrero et al., 1993). *In vivo* recordings with intracellular electrodes have been scarcely used due to technical difficulties related to mechanical stability; however, some remarkable studies have been published (Woolf et al., 1988). At present a few laboratories continue to use *in vivo* preparations with different electrophysiological approaches such as matrixes of electrodes which allow recordings from several neurons simultaneously (Greenspon et al., 2019). This technique opens the door to the study of circuits rather than single neurons.

*In vitro preparations* using rats or mice started to become popular toward the end of the last century, to solve the stability problem and to facilitate intracellular recordings. Intracellular current and voltage recordings are massively used but other techniques like extracellular electrode matrixes and calcium imaging are commonly reported coupled to *in vitro* preparations. Meanwhile, technologies based on novel voltage-sensitive dyes and genetically-encoded voltage indicators are currently under development (Hiyoshi et al., 2021). *In vitro* preparations include cell cultures as well as several *ex vivo* preparations developed for specific purposes. The entire or hemisected spinal cord preparations enable the use of simple techniques like dorsal root-ventral root recordings that proved very useful for pharmacological studies (Vicente-Baz et al., 2016). In addition, different slicing procedures have also been used to facilitate the access of a variety of electrodes to different laminae of the spinal cord. Besides sharp intracellular electrodes, electrodes for whole cell clamp, patch clamp and perforated patch electrodes are commonly used to study ionic currents and ion channels underlying the electrical behavior of dorsal horn neurons. Specialized preparations like the spinal cord attached to the hindlimb, tail, skin and brainstem have also been reported (King and Lopez-Garcia, 1993; Hachisuka et al., 2016; Lever et al., 2003). Under these *in vitro* conditions, stimulation protocols often include electrical stimulation of primary afferent pathways, intracellular current injection and more recently, optogenetics. Electrical stimulation of dorsal roots tends to recruit different afferent types in an intensity-dependent fashion so that thick myelinated Aβ fibers are activated by low-intensity currents whereas activation of thin myelinated Aδ and unmyelinated C afferents require progressively stronger stimuli. This procedure is simple and extensively used but does not allow for true selective activation of afferent types. Recently developed optogenetics and chemogenetics open the possibility of stimulating specific primary afferents or specific populations of secondary neurons contributing decisively to clarify the role of specific populations at shaping behavioral responses to somatosensory stimuli (Sharif et al., 2020; Harding et al., 2020; Choi et al., 2020).

Historically, there has been an interest in relating a neuron’s electrical behavior with its morphology, its position within the spinal cord, and its relationship to the rest of the somatosensory system. Intracellular and whole-cell recordings allow for injection of markers like biocytin or lucifer yellow, thereby enabling investigation of cell morphology at the end of an electrophysiological study (Grudt and Perl, 2002). Similarly, the use of genetic engineering in mice allows for the expression of green fluorescent protein or other fluorophores in specific populations of neurons, thereby allowing targeted electrophysiological studies (Hantman et al., 2004). Other imaging techniques include the use of anterograde markers to trace the destination of projecting axons, or retrograde markers to find the somas of neurons projecting to a particular brain region. These techniques have enabled electrophysiological studies of specific projection neurons (Li et al., 2023), which adds tremendous value to experimental observations that historically were performed blind to the identity of the recorded neuron.

## 3 Ionic currents involved in dorsal horn electrical activity

Dorsal horn neurons and the central terminals of primary afferents express a wide variety of ion channels representing all the main families reported in the CNS, which are briefly summarized in Table 1. Differences in the array of ion channels expressed by any individual neuron, and the relative balance between them, allow for an enormous variability in the electrical properties of a given neuron.

**TABLE 1:** Functional roles of the principal ionic currents expressed by dorsal horn neurons and their modulation in different animal pain models.

Ionic current/channels	Role in spontaneous activity	Function in evoked firing	Modulation in animal models of pain	References
I_Na_ persistent	Generation of rhythmic burst firing	Increases firing frequency, reduces AP adaptation, and promotes synaptic integration	Increase (spinal cord injury)	Li and Baccei, 2011; Lucas-Romero et al., 2018; Cho et al., 2015; Prescott and De Koninck, 2005; Lampert et al., 2006
I_NALCN_	Resting Na^+^ leak conductance Enhances spontaneous activity	Required for firing evoked by substance P	Increase (nerve injury, inflammation)	Ford et al., 2018; Zhang et al., 2021; Li and Baccei, 2021
I_H_	Generation of rhythmic activity	Regulates firing and input resistance Involved in rebound firing	HCN2 upregulation (oxaliplatin-induced neuropathy)	Rivera-Arconada et al., 2013; Rivera-Arconada and Lopez-Garcia, 2015; Liu et al., 2018
IK_A_	–	Modulation of neuron excitability Involved in delayed, gap and reluctant firing patterns	Altered voltage-dependent inactivation (inflammation) Reduction (capsaicin injection) Increase (LPS injection)	Hu et al., 2006; Melnick, 2011; Ruscheweyh and Sandkühler, 2002; Ruscheweyh et al., 2004; Graham et al., 2008; Rivera-Arconada and Lopez-Garcia, 2010; Zhang et al., 2018; Tadros et al., 2018
IK_DR_	Regulates spike duration	Involved in AP repolarization and fast AHP	Increase (inflammation)	Legendre et al., 1985
K_V_7 channels	–	Regulate resting membrane potential and promote adaptation in tonic firing neurons ^22^	–	Rivera-Arconada and Lopez-Garcia, 2005
IK_IR_ - GIRK channels (Kir3)	Resting K^+^ leak conductance; suppresses rhythmic bursting activity (Kir2) Decrease of rhythmic bursting	Generation of plateau potentials Contribute to adaptation in tonic firing neurons	Increase (neonatal surgical injury) Enhancement (inflammation, nerve injury)	Li et al., 2013; Ford and Baccei, 2016; Derjean et al., 2003; Li and Baccei, 2014; Santos et al., 2004; Ippolito et al., 2005
IK_Ca_	Promotes burst firing and AHP at burst termination	Involved in AHP. Decreases excitability and promotes adaptation in tonic firing neurons	Reduction (nerve injury)	Ma et al., 2023; Kim et al., 2008; Yang, 2016; Chen et al., 2009
I_CAN_	Facilitates pacemaker activity	Generation of plateau potentials	–	Morisset and Nagy, 1999

### 3.1 Sodium currents

Neurons located in the DH express different functional types of voltage-gated sodium currents with a particular spatial distribution. The axon hillock concentrates inactivating sodium channels, with this structure being fundamental for AP generation (Safronov et al., 1997). Tetrodotoxin-sensitive channels containing the Na_*v*_1.2 and Na_*v*_1.3 isoforms predominate in superficial areas of the DH (Hildebrand et al., 2011). Persistent sodium currents (I_*Na,P*_) have also been reported and may be important for the integration of synaptic inputs and the generation of spontaneous firing (Prescott and De Koninck, 2005; Li and Baccei, 2011; Lucas-Romero et al., 2018).

### 3.2 Potassium currents

Dorsal horn neurons express several types of potassium channels belonging to all known functional classes. Among the voltage-dependent channels, both inactivating and delayed rectifier currents are present in DH neurons (Wolff et al., 1998; Rivera-Arconada and Lopez-Garcia, 2010). For the inactivating type, the contribution of K_*v*_4.2 subunits may be of particular importance in pathological conditions (Hu et al., 2006). The sustained component may involve several different K_*v*_ subunits, including delayed rectifier and K_*v*_7/M-currents that can regulate the resting potential and the excitability of DH neurons (Murase et al., 1986; Nowak and Macdonald, 1983; Rivera-Arconada and Lopez-Garcia, 2005). Inward rectifier currents, as well as two-pore domain K^+^ channels, are also expressed by DH neurons and contribute to leak conductance (Li et al., 2013; Sano et al., 2003). Calcium-activated potassium currents of the SK and BK families are also present in DH neurons and modulate afterhyperpolarization and repetitive firing (Li and Baccei, 2011; Ma et al., 2023; Yang, 2016).

### 3.3 Calcium currents

Calcium currents expressed by DH neurons include transient and sustained types of voltage-gated calcium currents with different properties and pharmacology (Huang, 1989; Ryu and Randic, 1990), as well as persistent calcium currents (Prescott and De Koninck, 2005). For example, N-type calcium channels predominate in lamina I neurons, which also express L- and T-type currents (Heinke et al., 2004a). L-type currents are implicated in the acceleration and prolongation of firing upon depolarization in some DH neurons (Morisset and Nagy, 1998). T-type currents participates in burst firing and helps to adjust the firing after hyperpolarization (Sinha et al., 2021; Rivera-Arconada and Lopez-Garcia, 2015). Calcium entry is implicated in spontaneous firing of DH neurons and necessary for the regulation of calcium-dependent currents (Li and Baccei, 2011).

### 3.4 Mixed currents

Finally, other ionic currents are present in DH neurons, including the hyperpolarization-activated current (I_*h*_), which is responsible for the anomalous rectification in response to hyperpolarization and helps to set the resting membrane potential and adjust the timing of rebound firing (Rivera-Arconada et al., 2013; Rivera-Arconada and Lopez-Garcia, 2015; Yoshimura and Jessell, 1989a). The calcium-activated non-specific cationic conductance (I_*CAN*_) and the non-selective sodium leak channel (NALCN) mediate non-selective currents that are also expressed in DH neurons and may contribute to the regulation of neuronal excitability (Ford et al., 2018; Morisset and Nagy, 1999; Li and Baccei, 2011).

## 4 Spontaneous firing in the spinal dorsal horn

Spontaneous activity (SA) has been documented in both the superficial (Lucas-Romero et al., 2018; Li et al., 2013; Li and Baccei, 2011; Roza et al., 2016; Medrano et al., 2016) and deeper (Fernandes et al., 2018; McGaraughty et al., 2018; Quinn et al., 2010; Monteiro et al., 2006; Jiang et al., 1995) laminae of the DH, where it can be driven by the intrinsic membrane properties of the dorsal horn neurons and/or synaptic inputs activated in the absence of sensory stimulation. For example, 72% of neurons in the deep DH generated spontaneous action potentials triggered by suprathreshold EPSPs, resulting in a firing rate that ranged from 0.2 to 50 Hz (Woolf and King, 1989). While a high prevalence of SA was also observed in deep DH neurons receiving sensory input from the ankle and knee joints (Martindale et al., 2007), other studies reported that SA in the deeper laminae was rare in the absence of injury (Suzuki and Dickenson, 2006; Cata et al., 2006) and occurred at a relatively low (< 2 Hz) frequency (Palecek et al., 1992; Weng et al., 2003; Quinn et al., 2010; McGaraughty et al., 2018). A subset (4%) of deep DH neurons can reportedly undergo a rapid transition between low- and high-frequency modes of spontaneous discharge, which can be triggered by stimulation of their sensory receptive field (Monteiro et al., 2006). *In vitro* extracellular recordings from the mouse sacral spinal cord also revealed a relationship between the pattern of sensory input-evoked firing and the rate of SA (Thaweerattanasinp et al., 2016). Furthermore, the level of SA in the deep DH can be controlled in a cell type-dependent manner by the tonic release of neuromodulators, as suggested by the finding that blocking nitric oxide synthesis elevates SA in high-threshold mechanoreceptive neurons but not in the low-threshold mechanoreceptive population (Hoheisel et al., 2000).

While additional studies are needed to fully elucidate the degree to which the prevalence and rate of SA differ across distinct laminae of the spinal cord, available evidence suggests a similar frequency of SA between nociceptive neurons in lamina I and lamina V (Eckert et al., 2006). Nonetheless, prior *in vivo* extracellular recordings suggest that the presence of laminar differences may depend on the functional subtype of DH neuron. Notably, deep DH neurons that respond transiently to colorectal distension (CRD) exhibit a higher rate of spontaneous firing compared to their counterparts located in the superficial laminae, while those neurons that respond in a sustained manner to CRD (or are inhibited by CRD) display similar levels of SA across laminae (Ness and Gebhart, 1989). The use of multielectrode array (MEA) recordings in the rodent DH (Greenspon et al., 2019; Yu et al., 2017; Lucas-Romero et al., 2024) will undoubtedly facilitate a better understanding of the laminar differences in SA occurring within the DH network.

The observed patterns of SA have been variously described using terms which include irregular, regular, clock-like, rhythmic, bursting and pacemaker (Lucas-Romero et al., 2024) (see Figures 1B, C). Irregular fast-burst (IFB) neurons are characterized by brief, high-frequency bursts of action potentials (2–5 spikes at ∼100 Hz) that occur at irregular intervals (Roza et al., 2016). A particular case of the latter pattern is “double-spiking,” in which cells fire two action potentials (APs) with interspike intervals as low as 2 ms (Rojas-Piloni et al., 2007). Other DH neurons exhibit burst-firing at regular intervals and can be classified as either Regular Slow Burst (RSB) neurons, whose intraburst firing is either regular or irregular, or Regular Fast Burst (RFB) neurons which commonly display regular firing within the burst (Lucas-Romero et al., 2018). Pacemaker neurons previously described in the superficial DH (Li et al., 2013; Li and Baccei, 2011) likely correspond to the RSB subtype described above. Meanwhile, regular simple (RS) neurons, also known as clock-like (CL) neurons, discharge single APs at ∼6 Hz with regular interspike intervals (Roza et al., 2016; Luz et al., 2014).

Spontaneous firing can originate from synaptic activity or the intrinsic membrane properties of a given neuron. Notably, while antagonists of fast synaptic transmission (including blockers of AMPA and NMDA subtypes of glutamate receptors) abolish SA within irregular firing neurons, most neurons (∼82%) exhibiting a regular pattern of SA were found to be insensitive to these antagonists (Lucas-Romero et al., 2018; Luz et al., 2014). Similarly, the defining feature of pacemaker neurons is their intrinsic ability to generate rhythmic burst-firing, and therefore such bursting persists following the block of fast synaptic transmission both in the *ex vivo* spinal cord preparation (Li and Baccei, 2011) and in culture (Legendre et al., 1985). Pacemakers also exhibit a slow depolarization underlying the burst-firing which essentially endows the neuron with a bistable membrane potential (Li and Baccei, 2011) that is reminiscent of a subset of cells in the deep DH (Monteiro et al., 2006; Morisset and Nagy, 1998). Generally, DH neurons displaying SA exhibit a more depolarized resting membrane potential, a more hyperpolarized spike threshold and lower AP duration compared to neurons that lack SA (Lucas-Romero et al., 2018). Furthermore, pacemaker interneurons in the newborn superficial DH are characterized by a lower membrane capacitance and higher membrane resistance compared to adjacent, non-pacemakers in the same *ex vivo* spinal cord slices (Li and Baccei, 2011).

While SA has been documented in multiple cell types within the DH, including both excitatory and inhibitory interneurons residing in the superficial laminae (Li and Baccei, 2014), emerging evidence supports the existence of cell type-dependent variations in SA within the DH network. For example, within the neonatal spinal cord, SA is more common within lamina I projection neurons targeting the parabrachial nucleus (PB) compared to those innervating the periaqueductal gray (Li and Baccei, 2012), although *in vivo* recordings suggest that the level of SA within adult spinoparabrachial neurons is low (Bester et al., 2000). In addition, most pacemaker neurons in the newborn superficial DH correspond to glutamatergic interneurons located within lamina I, with an absence of intrinsic burst-firing within lamina II (Li and Baccei, 2011). This observation generally agrees with other work showing that rhythmic SA is more commonly found in local interneurons than spinal projection neurons (Sandkühler and Eblen-Zajjur, 1994; Fernandes et al., 2016). Nonetheless, recordings from lamina I projection neurons in an *ex vivo* intact spinal cord preparation revealed the existence of pacemaker activity in both the spino-PB and spino-PAG subpopulations (Li et al., 2015).

Interestingly, the ionic mechanisms underlying this intrinsic, rhythmic burst-firing also appear to vary across cell types. In contrast to lamina I interneurons (Li and Baccei, 2011), spinoparabrachial neurons showing pacemaker activity possessed higher membrane capacitance, lower membrane resistance, and a greater inward-rectifying K^+^ conductance compared to adjacent spinoparabrachial neurons that lacked intrinsic burst-firing (Li and Baccei, 2021). Age is also an important factor shaping the level of SA within the DH network, as the overall prevalence of SA and pacemaker activity is significantly downregulated between postnatal day (P)2 and P9 (Li and Baccei, 2011). Developmental alterations in passive membrane properties likely play a role, since superficial DH neurons show an age-dependent hyperpolarization of the resting potential and reduction in membrane resistance (Walsh et al., 2009). In addition, a developmental increase in the density of the rapid A-type voltage-gated K^+^ currents has been reported in the DH (Melnick, 2011). Finally, it should be noted that SA can also be synchronized across multiple subgroups of DH neurons, manifested as “population bursts” occurring at irregular intervals (Lucas-Romero et al., 2022), which can span several segmental levels in the lumbosacral spinal cord (Manjarrez et al., 2003).

While the above studies on SA in dorsal horn neurons were predominantly conducted using *ex vivo* electrophysiological recordings from spinal cord slices, the use of calcium imaging techniques has allowed investigators to document the existence of SA within the *in vivo* dorsal horn in both anesthetized (Sullivan and Sdrulla, 2022; Johannssen and Helmchen, 2010) and freely behaving (Sekiguchi et al., 2016; Shekhtmeyster et al., 2023) mice. The percentage of dorsal horn neurons exhibiting spontaneous Ca^2+^ transients in anesthetized mice *in vivo* is reportedly lower (5%) compared to prior estimates of SA obtained from spinal cord slices (62%; Doolen et al., 2012). The rate of spontaneous firing is significantly reduced by the use of anesthesia, with one study reporting a reduction from 0.52 to 0.08 Hz (Sekiguchi et al., 2016), which occurs in a dose-dependent manner (Sullivan and Sdrulla, 2022). Interestingly, there was no significant difference in the level SA between excitatory and inhibitory dorsal horn neurons regardless of the dose of anesthetic used (Sullivan and Sdrulla, 2022). While technical limitations have generally restricted such analysis to the superficial laminae of the spinal cord, recent methodological advances permitting the imaging of neuronal activity within deeper laminae (Shekhtmeyster et al., 2023) promise to further increase our understanding of the patterns of SA occurring within the complex dorsal horn network under both normal and pathological conditions.

### 4.1 Ion channels shaping spontaneous activity in the dorsal horn

Mounting evidence supports a key role for persistent, voltage-gated Na^+^ currents (I_*Na,P*_) in the generation of SA within the DH (Lucas-Romero et al., 2018; Li and Baccei, 2011; Cho et al., 2015). Interestingly, I_*Na,P*_ currents could be facilitated by reductions in external Ca^2+^ levels during repetitive firing, as previously reported in the ventral horn (Brocard et al., 2013; Tazerart et al., 2008). It is also clear that the level of leak (i.e., voltage-independent) membrane conductance profoundly regulates the level of SA within the DH network. Indeed, a high ratio of I_*Na,P*_ to leak conductance represents a hallmark feature of pacemaker neurons in the DH at P2–P3 (Li and Baccei, 2011). A major contributor to leak membrane conductance is the family of classic inward-rectifying K^+^ (K_*ir*_2) channels (Hibino et al., 2010). Blocking K_*ir*_2 channels in the neonatal DH unmasks rhythmic burst firing in ∼42% of non-bursting lamina I neurons (Li et al., 2013) and robustly enhances the firing of multiple subpopulations of lamina I projection neurons (Ford and Baccei, 2016). Activity-dependent elevations in the concentrations of extracellular K^+^ could also contribute to a reduction in constitutive K^+^ efflux during rhythmic burst-firing (Brocard et al., 2013). In contrast, tonic Na^+^ influx through NALCN channels, which are responsible for the vast majority of leak Na^+^ conductance in CNS neurons (Lu et al., 2007; Shi et al., 2016), constitutively enhances the spontaneous firing of lamina I spinoparabrachial neurons (Ford et al., 2018). A low level of resting Cl^–^ conductance could also facilitate SA in the DH (Keller et al., 2007), which can occur secondarily to weaker tonic synaptic inhibition mediated by GABA_*A*_R and glycine receptors (Takazawa and MacDermott, 2010). Furthermore, G protein-coupled inward-rectifying K^+^ (K_*ir*_3) channels (i.e., GIRK channels) are subject to tonic modulation by metabotropic glutamate (mGluR) and GABA_*B*_ receptors which can modulate endogenous burst-firing in the deep DH (Derjean et al., 2003).

L-type and N-type voltage-gated calcium channels (VGCC) are known to contribute to intrinsic burst-firing in the immature DH (Li and Baccei, 2011). Pacemaker activity can also be facilitated by the Ca^2+^-activated non-selective cationic current (I_*CAN*_) (Li and Baccei, 2011), which does not inactivate in the presence of elevated [Ca^2+^]_*int*_ (Partridge et al., 1994) and drives plateau potentials in other CNS neurons (Sheroziya et al., 2009). Meanwhile, although low-threshold T-type VGCC are dispensable for pacemaker activity in the neonatal DH (Li and Baccei, 2011), these channels are important for the generation of rebound depolarizations in deeper laminae (Rivera-Arconada and Lopez-Garcia, 2015). Hyperpolarization-activated cyclic nucleotide-gated (HCN) channels can cooperate with T-type VGCC to accelerate recovery from spike afterhyperpolarization and promote rebound firing (Li and Baccei, 2011; Rivera-Arconada and Lopez-Garcia, 2015) in addition to their contributions to leak conductance (Rivera-Arconada et al., 2013). Finally, multiple subtypes of voltage-gated K^+^ channels and Ca^2+^-activated K^+^ (K_*Ca*_) channels shape the different components of spontaneous burst-firing, including delayed-rectifier voltage-gated K^+^ channels which control spike duration and small-conductance K_*Ca*_ (i.e., SK) channels that are involved in burst termination (Li and Baccei, 2011; Legendre et al., 1985).

### 4.2 Functional significance of spontaneous activity within the dorsal horn network

It is well documented that SA contributes to neuronal survival as well as the formation and refinement of neuronal circuits throughout the CNS during early development (Shatz and Stryker, 1988; Tritsch et al., 2007). In the newborn superficial DH, it has been proposed that pacemaker activity could serve as a surrogate for noxious sensory experience by promoting the activity-dependent wiring of spinal nociceptive networks (Li and Baccei, 2011). The observation that pacemaker interneurons in the superficial DH connect to flexor and extensor motor pathways in the ventral horn (Li et al., 2015) raises the intriguing possibility that pacemakers might provide endogenous excitatory drive to developing sensorimotor networks that underlie nociceptive withdrawal reflexes. Meanwhile, the existence of intrinsic burst-firing in identified spinoparabrachial and spino-periaqueductal gray neurons (Li et al., 2015) suggests that pacemaker activity (or other forms of SA) could also provide endogenous glutamatergic drive to supraspinal pain circuits. Indeed, spontaneous firing in the DH has been linked to the generation of SA in the somatosensory cortex (Manjarrez et al., 2002), and spontaneous neuronal bursts have been observed in the human cortex during the neonatal period (Fabrizi et al., 2011).

## 5 Responses of dorsal horn neurons to natural stimuli

Classical studies performed in cats and rats *in vivo* allowed the study of DH neuron responses to natural stimulation of the skin and deep tissues. An in-depth discussion of this issue can be found elsewhere (Willis and Coggeshall, 2004; Price and Dubner, 1977). Many functional classifications were proposed for DH neurons but perhaps the most popular includes three types based on the nature of the stimulus driving the neuron (Mendell, 1966; Handwerker et al., 1975; Cervero et al., 1976). Low-threshold or type 1 neurons respond to light touch (or the electrical activation of thick myelinated afferents of cutaneous origin) and are found mostly in deep laminae. Wide dynamic range (WDR) or type 2 neurons respond to both touch and pinch of the skin and again are mostly reported in deep laminae but also in superficial LI. Finally, nociceptive-specific or type 3 neurons respond to nociceptive stimuli only (or the activation of nociceptive afferents from the skin, muscle or viscera) and are most commonly found in LI. Low threshold neurons tend to show little spontaneous activity, respond with brief trains of action potentials to natural stimuli such as touch and hair movement and may respond to cooling in the non-nociceptive range (Collins, 1984). Nociceptive specific neurons show also little spontaneous activity responding to intense mechanical stimulation of the skin and a proportion of them to noxious heating with a certain capacity for intensity coding within the nociceptive range (Christensen and Perl, 1970). In contrast, many wide dynamic range neurons tend to show spontaneous activity, non- or slowly-adapting responses to natural stimuli, and their firing frequency encodes stimulus intensity very well (Medrano et al., 2016; Wall et al., 1979). In addition to mechanical sensitivity, many dorsal horn neurons are sensitive to thermal stimuli including cooling and warming as well as noxious cold and heat. Some of these neurons are nociceptive-specific whereas others are of the WDR type (Gieré et al., 2021). Yet another class of neurons reported mainly in LII shows spontaneous activity that was inhibited by natural stimuli (Cervero et al., 1979).

An interesting concept emerging from these studies is that of “convergence,” by which one single DH neuron receives information from many primary afferents. In fact, careful examination of neuronal responses to specific classes of peripheral receptors, has allowed for identification of up to 19 different response profiles in WDR neurons, each one with a specific range of sensitivities (for example hair movement and pinch, or hair movement and pressure) (Heavner and De Jong, 1973). Additionally, intracellular studies demonstrate that neurons may display subthreshold responses to peripheral stimuli, further complicating the basic functional classification built on extracellular data (Woolf et al., 1988). Another derivative of the concept of convergence is that dorsal horn neurons have “receptive fields” with different sizes and shapes, but always larger than those of single afferents. Studies focusing on the properties of the receptive fields of these neurons demonstrated a somatotopic organization as well as the crucial influence of descending modulation which dynamically shapes their boundaries (Wall, 1967).

## 6 Firing patterns of dorsal horn neurons in response to intracellular current injection

Intracellular recordings allow for current injection through the electrode in the form of pulses, ramps or sinusoids, which can be used to transiently alter the membrane potential and elicit AP firing. This type of stimulation constitutes a widely used strategy in single neuron studies to test for basic electrophysiological properties (membrane resistance, capacitance, etc.) as well as the intrinsic excitability of neurons, understood as the ability to generate action potentials. Current injection enables the analysis of changes in electrophysiological properties of neurons due to certain treatments or to the application of drugs.

### 6.1 Firing patterns

**FIGURE 2:**
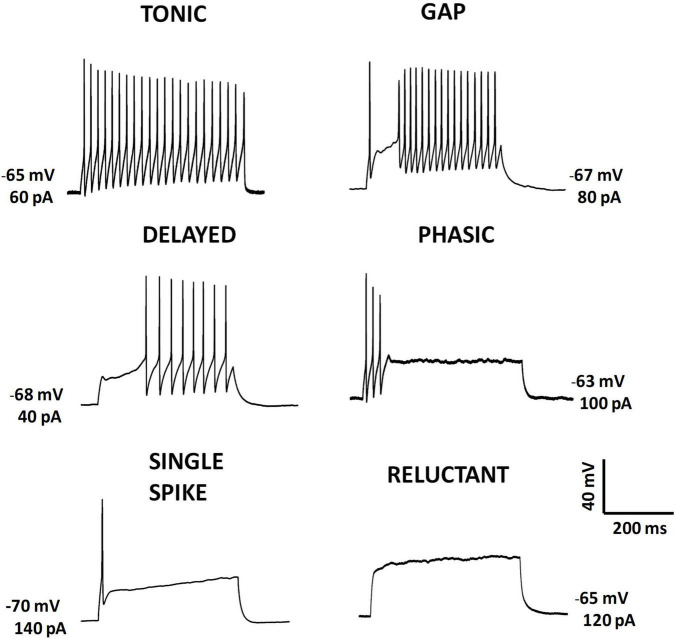
Action potential firing patterns evoked by intracellular current injection. Representative examples of firing patterns recorded from postnatal mouse lamina II neurons in *ex vivo* slice preparations. Action potentials were evoked by injecting depolarizing current steps using the patch-clamp technique. Initial membrane potentials and current step amplitudes are indicated near each panel.

Spinal cord neurons exhibit a wide variety of firing patterns. The most studied region within the spinal cord is probably the superficial DH (laminae I and II) because of its involvement in nociceptive signal processing, and most, if not all, of the documented firing patterns have been reported in neurons located within this area (see, for example, Grudt and Perl, 2002; Ruscheweyh and Sandkühler, 2002; Prescott and De Koninck, 2002). The firing pattern of action potentials in response to depolarizing current pulses has been widely used as a classification system, with responses to hyperpolarizing pulses also considered on occasion (Lopez-Garcia and King, 1994; Graham et al., 2004). A major classifying criterion is firing adaptation during current injection pulses (see Figure 2).

Tonic neurons are those that maintain the firing throughout the depolarization. Neurons that show firing adaptation with spikes restricted to the first part of the pulse have been referred to as phasic, initial bursting, transient or adapting firing neurons (Grudt and Perl, 2002; Ruscheweyh and Sandkühler, 2002; Prescott and De Koninck, 2002; Melnick et al., 2004a; but see Sinha et al., 2021). Perhaps an extreme case of adaptation could be the single spike pattern, where only one or two action potentials are evoked by depolarization. Temporary refractoriness to fire is the other basic feature for classification. Delayed firing neurons are those that show a delay in firing from pulse onset, gap firing neurons show periods of absence of firing after an initial discharge, and some neurons do not fire at all and are classified as reluctant firing neurons. In addition, some groups have extended the range of firing patterns (Grudt and Perl, 2002; Ruscheweyh et al., 2004) and defined special features associated to depolarization induced firing (e.g., plateaus; Morisset and Nagy, 1999). A more detailed study of firing patterns by analyzing firing latency allowed to Sinha et al. (2021) to separate delayed firing neurons into short and long-latency types. However, for some neurons the classification is not so straightforward. A mixture of firing characteristics can make the classification difficult. For example, some neurons may only fire a burst at the onset of low-amplitude pulses, and show sustained firing at large intensity pulses (e.g., bursting firing pattern from Ruscheweyh et al., 2004; initial bursting firing from Sinha et al., 2021). In addition, the expression of certain firing patterns is very dependent on the membrane potential at the initiation of the pulse (Ruscheweyh and Sandkühler, 2002).

Some studies have shown that excitatory neurons tend to show delayed firing, whereas tonic or initial burst firing is common in inhibitory neurons (Yasaka et al., 2010; Punnakkal et al., 2014; Heinke et al., 2004b). However other work has reported the opposite (Santos et al., 2007). Therefore, an accurate identification of neurotransmitter phenotype may require the analysis of additional features such as morphology (Grudt and Perl, 2002; Yasaka et al., 2010).

### 6.2 Ionic currents contributing to define the firing patterns

**FIGURE 3:**
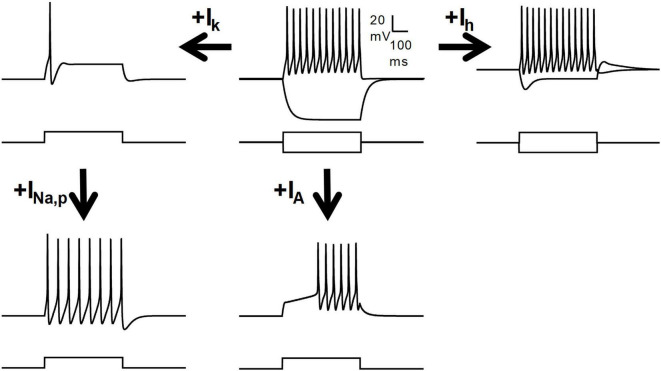
Regulation of the firing patterns by several voltage-dependent currents. Schematic drawing showing the influence of several subthreshold currents on the firing pattern of a model neuron. A tonic firing neuron may become adapting by the action of a slow subthreshold outward potassium current (I_k_), and can regain the tonic pattern by persistent sodium currents (I_Na_,_p_). The transient A-type potassium current (I_A_) is fundamental for the delayed firing pattern. The I_h_ may adjust the resting potential and determine the occurrence of pulse sag and rebound depolarization, but its role in defining the firing pattern is minor.

The expression of a particular firing pattern is dependent on the array of ionic currents expressed by a neuron (as summarized in Table 1). A small subset of defined ionic currents have been strongly related to the expression of specific firing patterns (Figure 3), but other additional channels are also important for finely shaping the output of a given neuron.

#### 6.2.1 Firing patterns related to A-type potassium currents

The A-type potassium current (I_*A*_) is the dominant conductance in the soma of delayed firing neurons (Melnick, 2011), whereas it is small or absent in tonic neurons (Ruscheweyh and Sandkühler, 2002; Melnick et al., 2004b). Blockade of I_*A*_ with 4-AP abolishes the delay in firing (Melnick, 2011; Ruscheweyh and Sandkühler, 2002). The presence of I_*A*_ with different kinetics can produce delayed firing with different latencies (Sinha et al., 2021). A slower I_*A*_ may be involved in gap firing. In addition, the proportion of reluctant versus delayed firing neurons may change with temperature and this difference may be explained by the sensitivity of I_*A*_ to the temperature (Graham et al., 2008).

#### 6.2.2 Ionic currents present in tonic firing neurons

Tonic firing neurons express slow-activating potassium currents of the delayed rectifier type (I_*KDR*_) (Melnick et al., 2004b). The K_*v*_7 channel opener retigabine hyperpolarizes deep DH neurons and promotes spike frequency adaptation in tonic neurons (Rivera-Arconada and Lopez-Garcia, 2005). Similarly, the activation of GIRK channels by the μ-opioid receptor in tonic neurons changes the tonic firing pattern to adapting (Santos et al., 2004). Tonic neurons can maintain firing even with a low percentage of inactivating sodium channels available (Melnick et al., 2004b) and also express persistent sodium currents (Prescott and De Koninck, 2005). Blocking persistent sodium currents with riluzole abolishes tonic firing and promotes adaptation (Lucas-Romero et al., 2018). Calcium-dependent potassium channels may also regulate the firing of tonic neurons. Activation of SK channels changes the pattern from tonic to adaptive, whereas its blockade with apamin increases firing frequency (Ma et al., 2023; Yang, 2016; Melnick et al., 2004b; Kim et al., 2008).

#### 6.2.3 Characteristics of ionic currents expressed by adapting firing neurons

Adapting firing neurons may express a lower density of both delayed rectifier potassium currents and inactivating sodium currents compared to tonic neurons, with the latter being a determinant of firing adaptation (Melnick et al., 2004a). T-type calcium currents have also been reported in a proportion of tonic neurons from lamina I and II, although their involvement in this firing pattern is not clear (Prescott and De Koninck, 2002; Wu et al., 2018; Candelas et al., 2019). In hamsters, neurons with strong adapting firing patterns, presumably single spike, showed large T-type currents (Ku and Schneider, 2011). Sinha et al. (2021) showed that initial bursting neurons consistently expressed T-type calcium currents. Hyperpolarization-activated currents (I_*h*_) may also be important for regulating firing. This current is widely expressed in DH neurons and its block can increase input resistance and firing (Lucas-Romero et al., 2018; Rivera-Arconada et al., 2013). The I_*h*_ is not specific for any firing pattern, but it has been reported more often in tonic and initial bursting neurons than in neurons displaying firing patterns implying the expression of A-type potassium currents (Hu et al., 2016; Yasaka et al., 2010; Boyle et al., 2019; Hughes et al., 2012; Zhu et al., 2021).

#### 6.2.4 Ionic currents responsible of plateau potentials

Dorsal horn neurons located in the superficial and deep dorsal horn may also produce voltage-dependent plateau potentials, consisting of prolonged depolarizations observed after the termination of the depolarizing current pulse injection in both tonic firing and adapting neurons (Morisset and Nagy, 1998; Fernandes et al., 2016). Generation of plateau potentials has been primarily linked to the expression of L-type calcium channels (Morisset and Nagy, 1999; Fernandes et al., 2016), together with the I_*CAN*_ current and K_*ir*_ channels (Morisset and Nagy, 1999; Derjean et al., 2003). The intense discharges and long-lasting after-discharges associated with plateau potentials represent an important intrinsic mechanism of input-output amplification, involved in the generation of wind-up (Morisset and Nagy, 2000).

### 6.3 Considerations in firing pattern analysis

The proportion of firing patterns under *in vitro* and *in vivo* conditions, as well as across different stages of postnatal development, is highly stable (Walsh et al., 2009; Baccei and Fitzgerald, 2005). The temperature is a factor that affect the kinetic of the ionic channels (for an exhaustive analysis on the effects of temperature on voltage dependent potassium currents see Ranjan et al., 2019), and hence temperature constitutes a potential factor that may influence the expression of firing patterns. Some reports have shown a changes in the distribution of firing patterns at different recording temperatures (Graham et al., 2008; Smith et al., 2015), but changing the temperature during the recording does not seem to affect the firing pattern (Santos et al., 2007), suggesting that differences may be due to a bias toward recording from certain types of neurons. The same is true when considering different orientations of the slices used for *ex vivo* recordings (Smith et al., 2015).

The firing pattern constitute an important parameter to characterize the excitability of dorsal horn neurons using a simple and reliable method, it is also a useful system for neuronal classification when applied under adequate conditions and considering additional features.

## 7 Firing induced by synaptic stimulation

Stimulation of PAFs, either by natural or electrical stimuli, results in the release of excitatory neurotransmitters, primarily glutamate. This, in turn, excites DH neurons, leading to the generation of excitatory postsynaptic potentials (EPSPs). Single or repetitive stimulation of glutamatergic synapses can produce suprathreshold mono- or polysynaptic responses, primarily mediated by AMPA and NMDA receptors. The firing pattern of APs in DH neurons depends on several factors, including the type and number of synaptic inputs, intrinsic properties of the neuron, recruitment of polysynaptic circuits, co-activation of inhibitory inputs, and ongoing neuronal activity.

**FIGURE 4:**
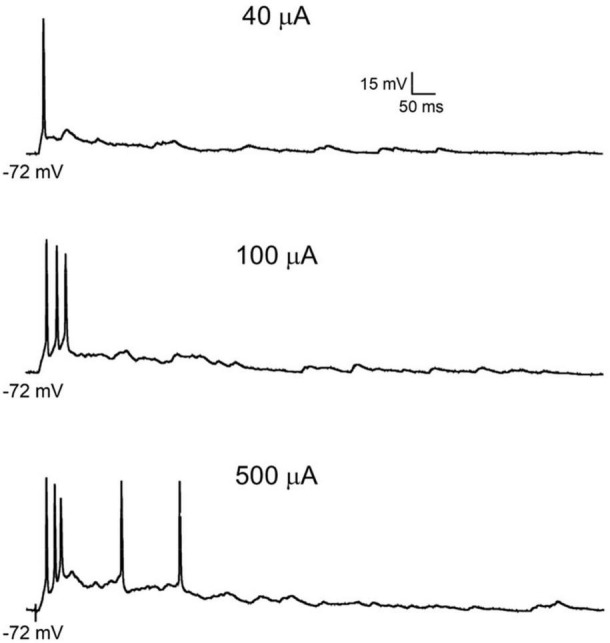
Primary afferent-evoked firing in rodent superficial dorsal horn neurons. Examples of action potential discharge in the same projection neuron shown in Figure 1B, in response to increasing intensities of dorsal root stimulation sufficient to recruit low **(top panel)** and high threshold fibers **(center and bottom panels)**.

In most interneurons located in lamina I and outer lamina II, single or repetitive electrical stimulation of nociceptive Aδ and C fibers in *ex vivo* preparations elicits suprathreshold EPSPs. Firing patterns can range from weak AP discharge to prolonged firing superimposed on slow depolarizations (Jeftinija, 1988; Yoshimura and Jessell, 1989b; Bardoni et al., 2000; see Figure 4). Lamina I projection neurons exhibit diverse responses to PAF stimulation. A study conducted on rat spinoparabrachial neurons identified three distinct groups of cells based on their responses to low-threshold fiber activation: (1) low-output neurons, typically responding with a single spike; (2) medium-output neurons (including both WDR and nociceptive-specific neurons), generating short AP bursts; (3) high-output neurons, firing prolonged AP discharges. Based on these properties, high output neurons, which represent only 20% of the spinoparabrachial neuron population, generate most of the output spiking activity (Agashkov et al., 2019).

Action potential firing can be induced in DH neurons not only by PAF stimulation, but also by activation of neighboring excitatory interneurons. Paired recording experiments in the rat isolated spinal cord have demonstrated that single stimuli delivered to lamina II excitatory interneurons evoke APs in approximately one-third of nearby postsynaptic neurons (Luz et al., 2010). In lamina I, excitatory inputs from neighboring neurons are sufficient to generate single or multiple spikes in about 50% of neurons projecting through the anterolateral system (Luz et al., 2010).

In the deep dorsal horn (laminae III-IV), most neurons receive low-threshold A fiber-mediated monosynaptic inputs, which elicit short bursts of APs. However, subpopulations of these neurons exhibit prolonged EPSPs with repetitive AP, likely due to the activation of polysynaptic circuits (King et al., 1988; Schneider, 2005). WDR neurons of the “antenna type,” located in lamina III and extending their dendrites to both superficial and deep DH, generate short AP bursts in response to low threshold inputs, while high-threshold C fibers evoke sustained firing (Fernandes et al., 2018). Similarly, WDR neurons in lamina V fire either a single spike or brief bursts in response to low threshold inputs, while nociceptive fibers tend to evoke long-lasting AP discharges that outlast the EPSP (Morisset and Nagy, 1998).

### 7.1 Modulation of firing by synaptic inhibition

Gamma-aminobutyric acid (GABA)- and glycine-mediated inhibitory postsynaptic potentials (IPSPs) are commonly observed in superficial DH neurons following stimulation of PAFs. These IPSPs arise from the activation of polysynaptic circuits involving inhibitory interneurons. In both the rat and mouse, these interneurons make up about 25% of the neurons in laminae I-II and 40% in lamina III (Polgár et al., 2013; Hughes and Todd, 2020). Recent studies have shown that in laminae I-III, about one third of inhibitory interneurons are exclusively GABAergic, while the remaining two-thirds release both GABA and glycine, with GABA being the predominant neurotransmitter. In deeper laminae, most neurons (about 90%) are also both GABA- and glycinergic, with a strong dominance of glycine (Miranda et al., 2022; Miranda et al., 2023).

Depending on the postsynaptic receptor involved (glycine or GABA_*A*_), IPSPs can exhibit fast or slow kinetics, respectively. Additionally, their latency can be short or long, in relation to the length of the polysynaptic chain involved (Luz et al., 2014; Yoshimura and Nishi, 1995). PAF-induced IPSPs exert different effects on AP firing: fast and/or short latency IPSPs can shunt monosynaptic EPSPs and limit their ability to generate APs, thereby preventing spike initiation; slow and/or long latency inhibitory responses may effectively prevent long-lasting repetitive firing and/or after-discharges (Yoshimura and Nishi, 1995). In addition to their role in mediating fast synaptic inhibition, GABA and glycine also mediate tonic inhibitory currents in the spinal cord DH (Takahashi et al., 2006; Takazawa and MacDermott, 2010; Gradwell et al., 2017). These tonic inhibitory currents modulate neuronal excitability by altering neuronal input resistance, action potential threshold, firing pattern, and input/output gain (Semyanov et al., 2004). Indeed, blocking GABA and/or glycine receptors in inhibitory interneurons has been shown to alter neuronal excitability by enhancing action potential discharge (Takazawa and MacDermott, 2010; Gradwell et al., 2017).

The degree of inhibition onto the postsynaptic neuron plays a crucial role in determining the involvement of specific glutamatergic receptors in EPSPs and subsequent firing. Fast EPSPs, evoked by single stimuli, primarily rely on AMPA receptors, including both Ca^2+^-permeable and Ca^2+^-impermeable subtypes (Luz et al., 2010; Tong and MacDermott, 2006; Yoshimura and Jessell, 1990; Santos et al., 2009). NMDA receptors can be recruited during repetitive stimulation of presynaptic inputs and prolonged postsynaptic discharges (Bardoni et al., 2000; Agashkov et al., 2019). The removal of inhibition through GABA_*A*_ and glycine receptor antagonists enhances the depolarization of lamina I and II neurons, leading to more robust activation of NMDA receptors. Consequently, this promotes the temporal summation of EPSPs, facilitates the generation of prolonged AP discharges, and activates reverberating polysynaptic circuits (Bardoni et al., 2000; Agashkov et al., 2019; Torsney and MacDermott, 2006).

In deep DH neurons, stimulation of low threshold PAFs results in inhibitory responses with variable latencies (Schneider, 2005). In a manner similar to what has been observed in the superficial DH, the removal of postsynaptic inhibition also increases neuronal excitability in laminae III-IV and promotes synaptic summation during repetitive stimulation (Betelli et al., 2015). In the same DH region, presynaptic GABA_*A*_ receptors expressed on A fiber terminals regulate afferent-induced firing by decreasing the number of APs evoked during repetitive A fiber stimulation (Betelli et al., 2015).

### 7.2 Modulation of firing by intrinsic membrane properties and spontaneous activity

Firing pattern analysis has been used as a simple method to infer how a given neuron might respond to a synaptic input, even though this stimulation does not reproduce the temporal characteristics of a real synaptic response (Graham et al., 2007). In an *ex vivo* preparation of the adult rat spinal cord, A-fiber electrical stimulation evoked APs only in tonic firing neurons (Thomson et al., 1989). In the hemisected cord-hindlimb preparation, WDR neurons often exhibited a tonic firing pattern in response to current injection, whereas neurons specific to tactile or nociceptive inputs displayed phasic patterns (Lopez-Garcia and King, 1994). In the whole spinal cord preparation, neurons firing with a phasic or single spike pattern responded to afferent stimulation with either a single spike or a short burst of APs. In contrast, tonic cells were more likely to generate prolonged EPSPs and sustained AP discharges (Fernandes et al., 2016; Agashkov et al., 2019; Dougherty and Chen, 2016). From a functional perspective, tonic-firing neurons that respond to PAF stimulation with prolonged EPSPs and firing could operate as integrators of synaptic inputs, faithfully transmitting sensory inputs in terms of duration and intensity. In contrast, rapidly adapting phasic neurons are optimally excited by short stimuli and do not rely on temporal summation for spike generation. Lastly, single spike neurons demonstrate the ability to follow high frequency trains, acting as precise coincidence detectors (Prescott and De Koninck, 2002).

Voltage-dependent ionic currents significantly influence the response to synaptic inputs in both excitatory and inhibitory interneurons. The A-type potassium current (I_*A*_) has been extensively studied in this context (reviewed in Clerc and Moqrich, 2022). Research on DH GAD67-GFP + (inhibitory) and GFP- (excitatory) interneurons in mouse spinal cord slices revealed that I_*A*_ densities are higher in GFP- cells compared to GFP + cells (Yoo et al., 2021). In excitatory neurons, a large I_*A*_ is necessary to reduce excitability under naïve conditions, whereas a small I_*A*_ in inhibitory interneurons leads to high excitability, aligning with the tonic firing pattern prevalent in these neurons (as described in section “8 Firing properties of identified subpopulations of dorsal horn neurons” and Table 2). In subpopulations of nociceptive excitatory interneurons, I_*A*_ prevents action potentials triggered by Aβ fiber stimulation in these neurons (Zhang et al., 2018). Blocking I_*A*_ with intracellular 4-aminopyridine permits Aβ fiber-mediated firing in nociceptive interneurons, resulting in the condition of allodynia (Zhang et al., 2018).

**TABLE 2:** Electrophysiological profiles observed in subpopulations of dorsal horn interneurons as identified by molecular markers.

Molecular marker	Primary afferents	Firing patterns	Characterized ionic currents	Neuronal class	Morphology	References
Vglut2	–	Delayed/Tonic (resting potential) Delayed (hyperpolarized potentials)	I_A_	Excitatory	Vertical (radial, central)	Maxwell et al., 2007; Yasaka et al., 2010; Yasaka et al., 2014; Punnakkal et al., 2014
GAD67	LI-II: C >> Aβ and Aδ	SDH: Tonic (immature) Phasic (adult) DDH: Tonic	–	Inhibitory	Islet (central, vertical)	Takazawa and MacDermott, 2010; Daniele and MacDermott, 2009; Punnakkal et al., 2014; Heinke et al., 2004b; Gassner et al., 2013; Punnakkal et al., 2014
GlyT2	Aβ > > Aδ > C	Tonic	–	Inhibitory	Islet (central, vertical)	Punnakkal et al., 2014; He et al., 2021
Somatostatin (SOM)	LII: Aβ and C > Aδ	Delayed (majority) Single spike	I_A_ I_CaT_	Excitatory (majority) (Inhibitory ∼5%)	Central, radial, vertical	Yasaka et al., 2010; Duan et al., 2014; Zhi et al., 2022
NPY receptor	Aδ and C	Phasic (resting potential) Delayed (hyperpolarized potentials)	I_A_ I_CaT_	Excitatory	–	Sinha et al., 2021
Substance P (SP)	–	Delayed (majority) Single spike, gap, phasic, reluctant	I_A_ I_h_	Excitatory	Radial	Dickie et al., 2019
GRP	–	Phasic/single spike (majority) Tonic, reluctant, delayed	I_A_ I_CaT_	Excitatory (majority) (inhibitory < 10%)	Central	Dickie et al., 2019
Calretinin (CR)	Inhibitory neurons: C >> Aδ > Aβ	Excitatory: delayed (majority), single spike Inhibitory: tonic (majority), phasic	I_A_ (excitatory neurons) I_h_ and I_CaT_ (inhibitory neurons)	Excitatory (majority) Inhibitory (minority)	Vertical, radial, central (excitatory) Islet (inhibitory)	Smith et al., 2015 Davis et al., 2023
**PKCγ**	Laminae I-IIo: HT Aδ and C Laminae IIi–III: Aβ and Aδ	Phasic (rat) Delayed, phasic (mouse)	–	Excitatory (majority) (Inhibitory < 5%)	Radial, central	Alba-Delgado et al., 2015; Abraira et al., 2017
GRP receptor (GRPR)	C > Aβ and Aδ	Delayed (majority) Tonic, phasic, single spike	I_A_ I_h_	Excitatory	Vertical	Bardoni et al., 2019 Polgár et al., 2023
NPFF	–	Delayed (majority) Tonic, phasic	I_A_	Excitatory	Vertical	Quillet et al., 2023
NMB receptor	**–**	Delayed (majority) Phasic, tonic, single spike	–	Excitatory (majority)	–	Wan et al., 2017
NKB (Tac2 gene)	Lamina II: C > Ad > Aδ Lamina III: Aβ > Aδ and C	Delayed (LII) Phasic (LIII)	–	Excitatory (majority)	–	Chen et al., 2020
Dynorphin (DYN)	Aβ, Aδ (HT C, LT C)	Phasic (neonatal) Tonic/Delayed (adult)	–	Inhibitory (majority) (excitatory ∼10%)	Vertical	Brewer et al., 2020; Duan et al., 2014
Parvalbumin (PV)	Inhibitory neurons: Aβ and LT Aδ	Tonic (majority) Phasic	I_h_ I_K(Ca)_	Inhibitory (majority)	Islet (majority), central	Boyle et al., 2019: Gradwell et al., 2022a; Gradwell et al., 2022b; Ma et al., 2023
Inhibitory nNOS/Galanin (PrP-GFP)	C > Aδ > Aβ	Tonic (majority) Phasic, single spike	–	Inhibitory	“Unclassified type”	Ganley et al., 2015
Cholinergic (ACh)	–	Tonic	–	Inhibitory	Islet	Mesnage et al., 2011
Early RET (Deep DH)	HT C + A fibers (“C type neurons”) A fibers only (“A type” neurons)	Tonic (majority) Delayed, phasic, single spike	–	Inhibitory	Islet (radial, vertical)	Cui et al., 2016
NPY	C > Aδ and Aβ	Tonic (majority) Intermittent bursting Tonic firing with gap	–	Inhibitory (majority)	Heterogeneous, not islet	Iwagaki et al., 2016

Finally, the processing of sensory transmission in the superficial DH can be significantly influenced by the ability of a subset of neurons to generate spontaneous intrinsic burst-firing. Recent work has demonstrated that adult mouse spinoparabrachial neurons displaying spontaneous burst-firing also exhibit higher levels of primary afferent-evoked AP discharge compared to those lacking such activity (Li et al., 2023). Additionally, the magnitude of afferent-evoked AP firing significantly correlates with the resting potential and membrane resistance, rather than the overall level of glutamatergic synaptic drive. This finding confirms the importance of the intrinsic membrane properties of DH neurons in shaping the input-output relationship at sensory synapses. In the deep DH, spontaneous bursting capability is associated with accelerated firing rates and enhanced spike afterdischarges (Morisset and Nagy, 1998; Russo and Hounsgaard, 1996). Nonetheless, the effect of spontaneous burst-firing on the gain of nociceptive signaling in the DH is likely to depend on the timing of the afferent input in relation to the phase of spontaneous activity, as pacemaker activity can filter out sensory inputs arriving during the generation of a spontaneous burst (Li and Baccei, 2011).

### 7.3 Short-term plasticity of dorsal horn neuronal excitability

Repetitive low-frequency stimulation of C-fiber inputs induces short-term synaptic facilitation, resulting in a progressive increase of the number of APs evoked by each stimulus, which eventually results in a prolonged after-discharge. This phenomenon, termed wind-up, was originally observed in lamina IV spinocervical neurons *in vivo* (Mendell and Wall, 1965; Mendell, 1966). Since then, numerous studies, both *in vitro* and *in vivo*, have characterized wind-up in various spinal neurons, including those in the superficial DH, deep DH, and motoneurons (for reviews, see Baranauskas and Nistri, 1998; Herrero et al., 2000). Experiments performed *ex vivo* on rat spinal cord have demonstrated that low-frequency activation of C fibers (0.2–2 Hz) is essential for inducing wind-up in DH neurons (Zieglgänsberger and Sutor, 1983; Jeftinija and Urban, 1994; Morisset and Nagy, 2000). In lamina I spinoparabrachial neurons, a subpopulation of excitatory interneurons contributes to wind-up by promoting reverberating network activity (Hachisuka et al., 2018). Under physiological conditions, synaptic inhibition reduces the activity of these polysynaptic circuits.

The generation of plateau potentials has been linked to the expression of wind-up in adult rats *in vivo* (Reali et al., 2011). Accordingly, wind-up is suppressed by blockers of L-type voltage-dependent calcium channels and I_*CAN*_, the two main depolarizing currents underlying plateau potentials (Morisset and Nagy, 2000; Fossat et al., 2007). Wind-up also involves a synaptic component, including both NMDA (Jeftinija and Urban, 1994; Dickenson and Sullivan, 1987; Woolf and Thompson, 1991) and tackykinin receptors (Baranauskas et al., 1995).

## 8 Firing properties of identified subpopulations of dorsal horn neurons

The extensive heterogeneity of firing patterns observed in DH neurons poses an obstacle to the use of this parameter to rigorously identify the neurotransmitter phenotype (i.e., glutamatergic vs. GABAergic) of the sampled neuron. The strongest association demonstrated to date is that inhibitory DH neurons commonly display a tonic pattern of repetitive AP discharge in response to prolonged depolarization, with no delay in the onset of firing and little evidence of spike frequency adaption or the expression of hyperpolarization-activated cation currents reported in the rat DH (Yasaka et al., 2010; Maxwell et al., 2007). Similarly, studies recording from GABAergic neurons in the immature mouse DH (identified via GAD67-EGFP expression) consistently reported a predominance of tonic firing within this population, with delayed and phasic patterns of discharge also observed to a lower extent (Takazawa and MacDermott, 2010; Punnakkal et al., 2014; Daniele and MacDermott, 2009). Meanwhile, GAD67-EGFP neurons in the adult DH commonly exhibited an initial bursting pattern of repetitive firing with a lower prevalence of tonic and gap firing observed (Heinke et al., 2004b), suggesting the possibility that age-dependent changes in the intrinsic firing properties of spinal GABAergic neurons may occur during later stages of development.

The close relationship between GABAergic neurons and tonic firing also appears to extend to the deep DH (Punnakkal et al., 2014; Gassner et al., 2013; Cui et al., 2016). In addition, glycinergic neurons residing in both the superficial and deep laminae of the DH (identified via the GlyT2 promoter) mostly show tonic firing (∼80%) with phasic discharge also reported (Punnakkal et al., 2014; He et al., 2021). In contrast, glutamatergic neurons in the DH (often identified via the expression of VGLUT2) commonly display a delayed firing phenotype, with gap and reluctant firing patterns also reported in this population, along with strong spike frequency adaptation (Yasaka et al., 2010; Maxwell et al., 2007; Yasaka et al., 2014). The prevalence of reluctant firing neurons within the population of excitatory neurons is in general agreement with prior observations that glutamatergic neurons generally possess higher rheobase levels and lower repetitive firing frequencies compared to inhibitory neurons in the DH (Punnakkal et al., 2014).

Firing patterns have also been shown to vary across different morphological subtypes of DH neurons, identified according to the location, size of the soma, and dendritic and axonal arborizations (Grudt and Perl, 2002). Islet cells commonly display tonic firing in response to persistent depolarization, which might be expected since they are predominantly inhibitory in nature (Yasaka et al., 2010; Maxwell et al., 2007). The relatively smaller subsets of GABAergic neurons showing other morphologies (such as radial and vertical) reportedly exhibit more variable firing properties (Maxwell et al., 2007). Meanwhile, other studies have demonstrated an association between tonic firing and a fusiform dendritic morphology, while pyramidal neurons in the DH often display phasic discharge and multipolar neurons exhibit both delayed firing and single-spiking patterns of repetitive firing (Prescott and De Koninck, 2002). Finally, vertical neurons (which include both excitatory and inhibitory subpopulations) commonly show delayed firing patterns, while transient central cells in the DH have been suggested to fire in a phasic manner (Yasaka et al., 2010; Yasaka et al., 2014; Lu and Perl, 2005). Little is known regarding the degree to which the firing properties of a given morphological cell type varies depending on laminar location within the DH.

### 8.1 Genetically identified subtypes of excitatory neurons

**FIGURE 5:**
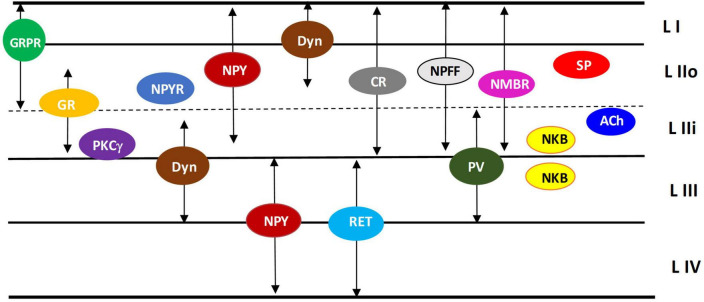
Localization of specific classes of neurons in the dorsal horn. Schematic diagram illustrating the localization of various neuronal classes described in section “8 Firing properties of identified subpopulations of dorsal horn neurons” and in Table 2 (refer to this table for abbreviations and references). Arrows indicate the regions where different neurons have been detected. The same class has been reported twice if two distinct subpopulations have been identified for the same neuron type, based on the their morphology and functional properties.

Glutamatergic neurons can be classified into distinct subpopulations based on their expression of biochemical markers such as neuropeptides (for review see Peirs et al., 2020), and the proliferation of available Cre mouse lines in recent years has afforded the opportunity to characterize the intrinsic firing properties of these discrete (albeit overlapping) subtypes of excitatory DH neurons (see Table 2 and Figure 5). For example, somatostatin-lineage neurons in the DH, which have been linked to the transmission of mechanical pain (Duan et al., 2014), predominantly show a delayed firing pattern (Yasaka et al., 2010) and discharge action potentials in response to Aβ-fiber or Aδ/C-fiber input depending on their dorsoventral location (Duan et al., 2014). Excitatory neurons expressing the Y1 receptor for neuropeptide Y (NPY), which have been implicated in neuropathic pain (Nelson et al., 2023), also predominantly show a delayed onset of firing in response to prolonged depolarization, which is accompanied by high expression of A-type voltage-gated K^+^ currents (Sinha et al., 2021). These cells also commonly exhibit rebound firing upon termination of membrane hyperpolarization (Sinha et al., 2021). Similarly, a high percentage (∼79%) of substance P-expressing DH neurons showed delayed firing and, as might be expected, expression of A-type K^+^ currents (Dickie et al., 2019). The calretinin-positive subpopulation of mouse DH neurons, which amplify nociceptive signaling in the DH and directly excite ascending projection neurons (Smith et al., 2019), also mostly showed a delayed firing pattern and large A-type K^+^ currents, with tonic and initial bursting patterns of repetitive firing also observed which may correspond to the small percentage (∼12%) of calretinin neurons that are predicted to be GABAergic (Smith et al., 2015). It should be noted that the calretinin population is not unique in being comprised of both excitatory and inhibitory interneurons, as this has also been observed in other neuronal populations including (but not limited to) those expressing nNOS, dynorphin and galanin (Sardella et al., 2011; Duan et al., 2014; Abraira et al., 2017). This complexity must be considered when attempting to associate biochemically- or genetically-identified subpopulations of DH neurons with a specific neurotransmitter phenotype.

Meanwhile, DH neurons expressing gastrin-releasing peptide (GRP), which have been implicated in spinal pruriceptive transmission, commonly display transient (∼50%) or single-spiking (∼33%) firing patterns (Dickie et al., 2019). Cells expressing the GRP receptor GRPR, which represent a subset of the postsynaptic targets of GRP-expressing neurons, also show a high prevalence of delayed firing (56%) with a sizeable number of cells displaying tonic (23%) and phasic (15%) firing also reported (Bardoni et al., 2019). GRPR+ neurons have also been shown to exhibit a high prevalence of single-spiking and correspond to vertical cells in terms of their morphology (Polgár et al., 2023). Another subpopulation of glutamatergic neurons implicated in itch signaling expresses the receptor for neuromedin B (NMBR) and features comparable numbers of delayed and phasic firing neurons (Wan et al., 2017). Finally, neurons expressing the tachykinin 2 (*Tac2*) gene (encoding neurokinin B) show a mix of delayed, tonic, initial burst and phasic firing within lamina II of the mouse DH, although *Tac2*+ cells in lamina III are nearly all phasic (Chen et al., 2020).

### 8.2 Genetically identified subtypes of inhibitory neurons

Like their glutamatergic counterparts, GABAergic neurons within the spinal DH can also be subdivided based on their expression of distinct biochemical markers, as summarized in Table 2 (for review see Peirs et al., 2020). Neurons derived from the prodynorphin (DYN) lineage, which suppress mechanical pain and itch (Duan et al., 2014; Kardon et al., 2014), predominantly exhibit tonic or delayed firing within the adult (P49-P63) superficial DH, while these cells show a high prevalence of phasic discharge during the neonatal period (P6-P7) (Brewer et al., 2020), thus highlighting the importance of considering age when attempting to relate the repetitive firing patterns to neurotransmitter phenotype. Meanwhile, parvalbumin-lineage neurons, which are important for the inhibition of mechanical sensitivity (Qiu et al., 2024), commonly exhibit islet or central morphology and reside in laminae II_*inner*_ – III, were found to be mostly tonic firing with some initial bursting also reported with a high incidence of I_*h*_ (Boyle et al., 2019; Gradwell et al., 2022a; Gradwell et al., 2022b). Interestingly, parvalbumin DH neurons can switch from tonic to phasic firing (with increased spike frequency adaptation) under neuropathic conditions, which likely contributes to the overall hyperexcitability of the spinal nociceptive network after peripheral nerve damage (Ma et al., 2023).

The subpopulations of neurons expressing nitric oxide synthase (nNOS) or galanin (identified via the expression of the prion promoter; PrP) predominantly showed tonic firing (∼72%) with initial burst-firing the next most prevalent pattern observed (Ganley et al., 2015). As expected from their GABAergic nature, cholinergic DH neurons (which partially overlap with the nNOS+ population) mostly exhibited tonic AP discharge with strong rebound firing (Mesnage et al., 2011). Similarly, genetic strategies that permitted the selective recording from the inhibitory subset of calretinin neurons in the DH showed these were overwhelmingly islet cells that fired in a tonic manner (Davis et al., 2023).

### 8.3 Intrinsic firing properties of ascending spinal projection neurons

Despite their glutamatergic nature, extrapolating the above findings to the output neurons of the spinal nociceptive circuit is difficult given that projection neurons comprise less than 5% of the overall neuronal population within the spinal DH (Spike et al., 2003; Cameron et al., 2015) and thus are commonly identified via retrograde labeling from the brain, although the recent development of Phox2a-Cre mice allows for the genetic identification of a subset of ascending spinal projection neurons (Roome et al., 2020). Rat spinoparabrachial and spino-PAG neurons show almost exclusively tonic firing during early life (i.e., P2–P5) but phasic, delayed and bursting patterns of repetitive discharge become evident by adolescence (P30–P32) (Li and Baccei, 2012). Other studies conducted in the rat DH reported that the gap and bursting firing patterns were mostly restricted to projection neurons (as opposed to unidentified DH neurons) and were commonly found in the spinoparabrachial and spino-PAG populations, respectively (Ruscheweyh et al., 2004). The gap and bursting firing patterns were attributed to the expression of A-type K^+^ currents and T-type voltage-gated Ca^2+^ currents, respectively (Ruscheweyh et al., 2004). Meanwhile, adult mouse spino-PAG neurons show spontaneous burst-firing (as well as irregular discharge) and predominantly displayed tonic or initial burst patterns of firing in response to prolonged current injection, with spontaneously bursting cells also showing high levels of primary afferent-evoked firing (Li et al., 2023).

Other studies revealed that mouse spinoparabrachial neurons can be clustered into four distinct subpopulations based on their electrophysiological properties, and those neurons located in the deep DH (i.e., laminae III-V) exhibited higher repetitive firing frequencies compared to the lamina I spinoparabrachial population (Browne et al., 2021). In comparison to interneurons in the DH, projection neurons are reportedly more excitable compared to unidentified cells (> 95% of which are expected to correspond to interneurons) which showed mostly tonic firing (Graham et al., 2008).

## 9 Changes in neuronal activity induced by chronic pain

There is mounting evidence indicating that peripheral lesions can change the electrical behavior of DH neurons. Peripheral lesions may affect the skin, muscles, joints or viscera leading to inflammation as well as lesions affecting sensory nerves that lead to neuropathy. Central changes in nociceptive processing are usually referred to as central sensitization (Woolf, 1983), a phenomenon different from (and dependent on) peripheral sensitization. Central sensitization occurs as a consequence of the arrival of a sustained barrage of action potentials to the spinal cord and the brain through nociceptive pathways.

**FIGURE 6:**
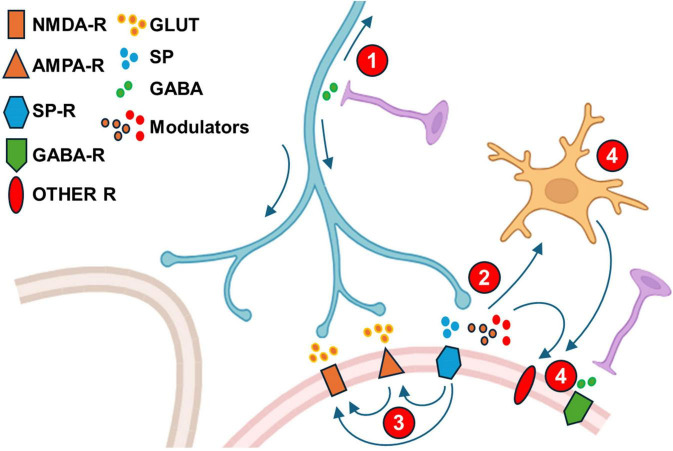
Modifications of synaptic transmission following peripheral injury. Cartoon depicting synaptic changes in dorsal horn occurring following peripheral injury. Role of descending system excluded for clarity. See main text for details. (1). Enhancement of presynaptic primary afferent depolarization and subsequent firing in orthodromic- and antidromic directions plus facilitation of conduction across branching points (arrows); (2). Presynaptic release of mediators by afferents following an NGF phenotipic change of primary afferents (Substance P, BDNF, ATP, CCL2, CSF1); (3). Enhancement of excitatory aminoacid receptor function (AMPAR and/or NMDAR following activation of other receptors (Neurokinin receptors and others). (4). Neuron-glia interactions: Microglia activation by inflammatory mediators (prostaglandins) and mediators released from primary afferents (ATP, CCL2, CSF1). (5). Modulators from glia and primary afferents (like BDNF) or byproducts of inflammation (Prostaglandins) act on second order dorsal horn neurons to depress or alter inhibitory transmission.

Many observations using different preclinical models have established that nociceptive DH neurons, particularly wide dynamic range neurons, can increase their spontaneous firing, responses to natural stimuli and the size of their receptive fields under conditions of persistent pain. For example, a recent study using a model of chemotherapy-induced neuropathic pain shows that WDR neurons acquire spontaneous activity and a wider sensitivity to stimuli of different modalities (Meesawatsom et al., 2020). In addition, some of these neurons generate post-discharges to innocuous stimuli. Similarly, observations from a model of joint inflammation show that DH neurons enhance their preexisting responses, lower their response threshold, and expand their receptive fields, responding even to movement in the contralateral joint (Neugebauer and Schaible, 1990). The behavior of high-threshold neurons is also affected by peripheral lesions in similar ways (Hylden et al., 1989). It is interesting to note that different pain models tend to produce central changes with particular traits (Zain and Bonin, 2019). Different mechanisms are under discussion to explain changes in the DH that follow a peripheral injury. At the cellular level, these mechanisms can be reduced to synaptic plasticity and alterations in intrinsic membrane excitability (see Figure 6). Synaptic and intrinsic mechanisms are not mutually exclusive, and in fact may interact to generate the final hyperalgesic phenotype (Rivera-Arconada and Lopez-Garcia, 2010).

### 9.1 Synaptic changes in the dorsal horn following peripheral injury

The synapses between primary afferents and DH neurons have been under intense scrutiny because they are the first relay of sensory information. These synapses can be regulated at the presynaptic level to modulate neurotransmitter release from primary afferents or at the postsynaptic level to modulate receptor sensitivity.

A presynaptic mechanism discussed at large in the literature is that of primary afferent depolarization (PAD). PAD was first described as a physiological means to produce presynaptic inhibition in muscle afferents (Eccles et al., 1962). PAD is produced by release of GABA and subsequent depolarization due to the outward movement of chloride from the afferent terminals which contain relatively high internal Cl^–^ levels. Inflammatory stimuli can enhance PAD to reach firing threshold, thereby converting an inhibitory mechanism to an excitatory one (Pitcher and Cervero, 2010). Action potentials generated at central branches of the afferents are then transmitted antidromically to contribute to peripheral neurogenic inflammation and orthodromically to produce allodynia (Willis, 1999). Recent data suggest that PAD may also serve to facilitate AP conduction through branching points at central terminals of primary afferents (Hari et al., 2022). Thus, inflammation-induced enhancement of PAD could lead to increased excitation in the DH and enlargement of receptive fields.

Experimental evidence shows that synchronous firing of DH neurons associated with activity in primary afferents increases significantly after inflammation (Lucas-Romero et al., 2022). Interestingly, population bursts in the DH (i.e., the synchronous firing of multiple neurons) have been related to the generation of PAD and the backfiring of sensory neurons (Lucas-Romero et al., 2022), which are important for gating sensory inputs in the spinal cord (Boyle et al., 2019) as well as promoting the peripheral release of neuropeptides and subsequent neurogenic inflammation (Willis, 1999). Other presynaptic mechanisms include phenotypic changes in primary afferents. Substance P release may be increased under inflammatory conditions as a result of peripheral NGF-induced phenotypic modification of primary afferents (Woolf, 1996). Other phenotypic changes, associated mainly with neuropathy, have been reported to include the release of CCL2 and CSF1 by primary afferents (Gosselin et al., 2005).

Amino acid-mediated transmission has been intensively studied in connection with central sensitization. Changes in NMDA receptors on their own, or in combination with changes in subunit composition of AMPA receptors, may work as final effectors to increase excitability of DH neurons in inflamed animals by increasing postsynaptic glutamate-induced depolarizations and allowing calcium entry into neurons that promote further changes in excitability (Park et al., 2009). Many other molecules may modulate excitability of DH neurons through interaction with glutamate receptors. For example, dynorphin, substance P and CGRP released from nociceptive primary afferents may trigger intracellular signals that impinge on NMDA receptors to generate hyperexcitability of DH neurons (Dubner and Ruda, 1992; Bird et al., 2006).

Inhibitory synapses mediated by GABA and glycine are also subjected to plasticity during inflammatory and neuropathic conditions. Key mediators of inflammation like prostaglandin E2 have been shown to increase excitatory transmission and block inhibitory transmission mediated by glycine in DH neurons (Zeilhofer, 2008). A loss of inhibition through GABAergic synapses has been reported in models of neuropathic pain as well (Coull et al., 2003). This mechanism involves downregulation of KCC2 in superficial dorsal horn neurons which in turn causes a shift in the reversal potential of chloride ions such that GABA can have a depolarizing effect. A decrease of the spinal inhibitory tone, which frequently occurs following nerve injury, could enhance the activity of the excitatory network, giving rise to the abnormally elevated wind-up that is observed in some pathological conditions (Herrero et al., 2000).

Descending systems can release serotonin, noradrenaline and GABA from axons originating in brainstem nuclei. Monoamines interact with a variety of membrane receptors located at central terminals of primary afferents and/or DH neurons to produce either inhibitory or excitatory effects on nociceptive transmission via presynaptic and postsynaptic actions (Lopez-Garcia, 2006). Following peripheral inflammation, excitatory effects may be increased (Carr et al., 2014) and/or inhibitory effects may be decreased (Cervero et al., 1991) to cause an imbalance between excitation and inhibition.

Neuroinflammation and the intervention of glial cells constitutes a novel player in central sensitization related to both inflammatory and neuropathic pain as well as diffuse chronic pain syndromes similar to fibromyalgia. CCL2 and CSF1 together with ATP released from primary afferents activate microglia which in turn release proinflammatory interleukins and probably BDNF (Gosselin et al., 2005; Denk et al., 2016). BDNF released from microglia or primary afferent neurons has been shown to increase NMDA-mediated responses (Kerr et al., 1999), induce the anion shift in dorsal horn neurons (Coull et al., 2005), evoke micro-structural changes in synaptic elements (Bareyre et al., 2002) and promote other complex changes in neuronal metabolism (Pezet et al., 2002) which may contribute to the maintenance of hyperalgesia.

### 9.2 Modifications in intrinsic excitability and ion channel expression following peripheral injury

Prior studies comparing the basic electrophysiological properties of spinal neurons in naïve (or sham) vs. neuropathic animals reported no differences in resting potential, membrane resistance or in the proportion of neurons with different firing patterns (Gassner et al., 2013; Balasubramanyan et al., 2006; Schoffnegger et al., 2006). However, recent work reveals subtle changes in the excitability of inhibitory parvalbumin neurons in the DH. Tonic-firing parvalbumin neurons located ipsilateral to the lesion may require a higher current amplitude to maintain tonic firing and produce a lower firing frequency compared to neurons on the contralateral side in response to the same current injection (Boyle et al., 2019). Alternatively, these neurons may undergo a change in firing pattern from tonic to adaptive after nerve lesion (Ma et al., 2023). These differences in excitability could be relevant for the expression of allodynia after neuropathy. In this line, the neonatal treatment with the chemotherapeutic drug vincristine enhance the excitability of spinoparabrachial neurons located in lamina I (Schappacher et al., 2019). However, in a model of chronic spinal cord injury, inhibitory lamina I neurons with tonic and initial burst patterns show higher excitability as demonstrated by a depolarized membrane potential, lower rheobase and higher firing frequency. This mechanism may serve as a compensatory homeostatic response to the state of hyperexcitability in DH circuits (Dougherty and Hochman, 2008).

Changes suggestive of a reduced excitability of spinal neurons have also been reported in different animal models of pain (Li and Baccei, 2014; Farrell et al., 2017). Certain types of excitatory and inhibitory neurons show a lower membrane resistance and more hyperpolarized resting potentials, together with reduced firing frequency, in adult animals subjected to paw incision at 3 days after birth (Li and Baccei, 2014). In a model of colitis, more hyperpolarized membrane potentials and an alteration in AP firing adaptation have been described in certain DH neurons (Farrell et al., 2017). In a model of paw inflammation, an increase in excitability in the early stages of the inflammatory process was followed by a decrease in the longer term (Rivera-Arconada and Lopez-Garcia, 2010). In a recent report, the axon initial segment has been shown to shift distally away from the soma in inhibitory neurons after peripheral inflammation, which could be responsible for decreased excitability (Caspi et al., 2023).

Most of the studies highlighted above show that the excitability of spinal neurons may be altered in specific cell types during pathological processes. The mechanisms responsible for these changes are not fully understood, although changes in the expression of genes encoding sodium, calcium and potassium channels have been shown under pain states (Yang et al., 2004) (see Table 1). An increase in delayed rectifier currents could be responsible for a reduction in firing frequency during inflammation (Rivera-Arconada and Lopez-Garcia, 2010). Transient potassium currents (A-type current) may also be important in models of inflammation. Alterations in the voltage-dependent inactivation of this current may contribute to increased excitability in the early stages of an inflammatory process (Rivera-Arconada and Lopez-Garcia, 2010). Following capsaicin injection, this current may be reduced, allowing normally subthreshold inputs from Aβ fibers to reach firing threshold in neurons located in the superficial DH (Zhang et al., 2018). Interestingly, an enhancement of A-current may serve as a compensatory mechanism to reduce excitability once potentiation of synaptic strength has been established following the lesion (Tadros et al., 2018). Inward-rectifier potassium currents can also be altered after a neonatal insult thereby decreasing spinal neuronal excitability (Li and Baccei, 2014). Other potassium channels may also change their expression or modulation in models of neuropathy and inflammation (BK: Chen et al., 2009; Kir3: Ippolito et al., 2005), but in other studies no changes have been found (Mongan et al., 2005).

Regarding voltage-gated sodium channels, changes in the expression of alpha and beta subunits have been shown in models of neuropathy (Blackburn-Munro and Fleetwood-Walker, 1999; Hains et al., 2004; Lampert et al., 2006). Leak sodium channels are increased in both inflammatory and neuropathic models and are important regulators of membrane potential and excitability (Zhang et al., 2021; Li and Baccei, 2021). Meanwhile, increased activity of L- and N-type calcium channels has been reported in an animal model of streptozotocin-induced diabetes (Voitenko et al., 2000). In models of neuropathy caused by nerve injury, Ca_*v*_1.2 and Ca_*v*_1.3 channels (L-type) are up- and downregulated, respectively (Dobremez et al., 2005). These changes can be related to an increase in the expression of plateau potentials, but they may also be involved in both the reduction of wind-up in wide dynamic range neurons due to the decrease in Ca_*v*_1.3 channels and the hyperexcitability of spinal neurons due to the increase in Ca_*v*_1.2 (Reali et al., 2011; Radwani et al., 2016). Alterations in N-type channels may be involved in sensitization at the presynaptic level, with both increases and decreases described depending on the injury model (Cizkova et al., 2002; Rycroft et al., 2007). Changes in the expression of the α2/δ1 regulatory subunit have also been described and may contribute to increased calcium currents in models of peripheral neuropathy (Li et al., 2004; Alles et al., 2018) and chemotherapy-induced neuropathic pain (Chen et al., 2019; Xiao et al., 2007; Gauchan et al., 2009). Finally, peripheral lesion has been shown to regulate expression of several other channels. For example, the CNGA3 subunit of cyclic nucleotide-gated channels (Heine et al., 2011), HCN2 channels (Liu et al., 2018) and acid-sensing ion channels (Wu et al., 2004; Duan et al., 2007) are upregulated in DH neurons and their contribution to nociceptive processing is under investigation.

The modulation of ion channel expression provides a powerful mechanism to regulate the excitability of DH neurons by adjusting their input-output relationship. Changes directed to increase the excitability have been reported, but homeostatic compensation may also have a profound influence on the processing of nociceptive information.

## 10 Concluding remarks and future perspectives

In this review, we have described a wide range of activity patterns associated with both spontaneous and evoked action potential firing in the superficial and deep DH. This wide heterogeneity likely reflects the large variety of neuronal subtypes present in the DH.

A molecular characterization of the different DH populations has recently been provided by several studies employing RNA sequencing along with other molecular and functional approaches. By using the single-cell RNA sequencing technique, Häring et al. (2018) identified 15 excitatory and 15 inhibitory molecular subtypes located in the mouse DH. More recently, using single-nucleus RNA sequencing (snRNA-seq), five distinct populations of projection neurons belonging to the anterolateral system have been described (Bell et al., 2024). These neuronal clusters were found in specific locations within the mouse DH laminae and exhibited different functional roles in processing pain, itch, and thermal stimuli. Similarly, the use of snRNA-seq, spatial transcriptomics, and immunohistochemistry in the adult human spinal cord has enabled the identification of 64 different cell clusters (29 of glial cells and 35 of neurons), organized primarily by anatomical location (Yadav et al., 2023).

Hierarchical cluster analysis combining electrophysiological and morphological properties has revealed the presence of 5 relatively distinct clusters in the mouse superficial DH: two groups of excitatory interneurons and three groups of inhibitory interneurons (Browne et al., 2020). Interestingly, electrophysiological properties more effectively distinguished excitatory and inhibitory phenotypes than morphological-based clustering, showing that inhibitory interneurons are generally more excitable, tend to fire in a tonic pattern, and have a weak excitatory drive. Conversely, excitatory interneurons are typically less excitable, commonly exhibit delayed and phasic firing profiles, and are subject to a strong excitatory synaptic drive.

The molecular characterization of DH neurons, combined with the study of animal behavior, provide important insights for targeting specific neuronal populations and understanding their functional role in sensory processing. However, this classification alone is insufficient to differentiate the numerous subtypes of DH neurons. Given that electrophysiological, morphological, functional (excitatory vs inhibitory; interneuron vs. projection neuron), and molecular traits often do not cluster together, and that none of these single properties can predict the others within a single neuron, more complex analytic procedures should be employed. These procedures should include a comprehensive collection of electrophysiological characteristics, identification of morphological and molecular traits, analysis of excitatory and inhibitory inputs, assessment of neuronal connectivity, and evaluation of the functional role of the neuron in nociceptive behavior. The Patch-seq technique, which integrates electrophysiological, transcriptomic, and morphological characterization of individual neurons, could provide significant insights into understanding the functional properties of DH neurons. Moreover, to classify the subpopulations of DH neurons that play unique roles in somatosensation, the analysis should also consider other important variables, such as animal species, age, sex, and the specific pain models employed. Taking all these factors into account, it becomes clear that each DH neuron operates as an individual entity, adjusting and modulating its activity in response to the functional state of the DH network.
